# Increasing Dose of Autologous Bone Marrow Mononuclear Cells Transplantation Is Related to Stroke Outcome: Results from a Pooled Analysis of Two Clinical Trials

**DOI:** 10.1155/2016/8657173

**Published:** 2016-07-21

**Authors:** Francisco Moniche, Paulo Henrique Rosado-de-Castro, Irene Escudero, Elena Zapata, Francisco Javier de la Torre Laviana, Rosalia Mendez-Otero, Magdalena Carmona, Pilar Piñero, Alejandro Bustamante, Lucía Lebrato, Juan Antonio Cabezas, Alejandro Gonzalez, Grabriel R. de Freitas, Joan Montaner

**Affiliations:** ^1^Department of Neurology, Hospital Universitario Virgen del Rocío, 41013 Seville, Spain; ^2^Instituto de Biomedicina de Sevilla-IBiS, Hospital Universitario Virgen del Rocío, 41013 Seville, Spain; ^3^Instituto de Ciências Biomédicas, Federal University of Rio de Janeiro, 21044-020 Rio de Janeiro, RJ, Brazil; ^4^Instituto de Biofísica Carlos Chagas Filho, Federal University of Rio de Janeiro, 21044-020 Rio de Janeiro, RJ, Brazil; ^5^Department of Hematology, Hospital Universitario Virgen del Rocío, 41013 Seville, Spain; ^6^Department of Radiology, Hospital Universitario Virgen del Rocío, 41013 Seville, Spain; ^7^Neurovascular Research Laboratory, Institut de Recerca Vall d'Hebron, Hospital Vall d'Hebron, 08035 Barcelona, Spain; ^8^D'Or Institute for Research and Education, 22281-100 Rio de Janeiro, RJ, Brazil

## Abstract

*Background and Purpose.* BM-MNC transplantation improves recovery in experimental models of ischemic stroke. Clinical trials are ongoing to test efficacy in stroke patients. However, whether cell dose is related to outcomes is not known.* Methods*. We performed a pooling data analysis of two pilot clinical trials with autologous BM-MNCs transplantation in ischemic stroke patients. Cell dose and route were analyzed to evaluate their relation to good outcome (m-Rankin scale [mRS] score 0–2) at 6 months.* Results*. Twenty-two patients were included. A median of 153 × 10^6^ (±121 × 10^6^) BM-MNCs was injected. Intra-arterial route was used in 77.3% of cases. A higher number of cells injected were associated with better outcomes at 180 days (390 × 10^6^ [320–422] BM-MNCs injected in those patients with mRS of 0–2 at 6 months versus 130 × 10^6^ [89–210] in those patients with mRS 3–6, *p* = 0.015). In the intra-arterially treated patients, a strong correlation between dose of cells and disability was found (*r* = −0.63, *p* = 0.006). A cut point of 310 × 10^6^ injected cells predicted good outcome with 80% sensitivity and 88.2% specificity.* Conclusions*. Similar to preclinical studies, a higher dose of autologous BM-MNC was related to better outcome in stroke patients, especially when more than 310 × 10^6^ cells are injected. Further interventional studies are warranted to confirm these data.

## 1. Introduction

Stroke is one of the leading causes of morbidity and long-term disability in the world, with about one-third of survivors being permanently disabled [1]. In the very acute phase of stroke thrombolytics and endovascular thrombectomy can reduce stroke disability; however, there are few options for recovery once the neurological deficits are established. In recent years, extensive cell therapy preclinical research has demonstrated a neurorestorative effect after cerebral ischemia, improving neurological outcomes even in the long term [2–4]. Amongst the most promising are bone marrow mononuclear cells (BM-MNCs), which have consistently demonstrated efficacy in animal stroke models in different laboratories and species [4–6]. These cells have the advantage of being rapidly isolated from bone marrow, do not require culture, and can be injected within hours from bone marrow aspiration and therefore suited for autologous administration even in the acute phase of stroke. In the last years, some preliminary phase I/II trials have shown the safety and feasibility of autologous bone marrow transplantation in stroke patients [7–11]. However, many questions regarding dose, route, and type of cells need to be addressed before starting phase III trials.

A wide range of cells numbers has been used for transplantation in animal stroke models and in clinical trials. While in preclinical studies there is strong evidence that a higher dose of cells increases the probability of a good neurological outcome [12, 13] the optimal number of cells to be transplanted for ischemic stroke is largely unknown. This raises the question of whether a higher dose of BM-MNCs will produce a greater effect in recovery in stroke patients.

In order to evaluate the relation of dose and recovery of neurological deficit in stroke patients treated with BM-MNCs, we performed a pooling data of clinical trials with autologous BM-MNCs transplantation.

## 2. Material and Methods

### 2.1. Pooled Clinical Trials

We combined individual data from two different pilot phase I/II clinical trials, which were designed to assess the safety and feasibility of BM-MNCs transplantation in ischemic stroke patients and conducted in Spain and Brazil [7–9]. Thirty-two patients were included in both clinical trials. Of them, ten patients were controls and twenty-two patients were actively treated with BM-MNCs. Patients included had an ischemic stroke in the MCA territory. Inclusion and exclusion criteria were largely similar between the two trials. Main differences included different time windows, with patients treated in the Spanish trial within 5–9 days of stroke onset and those treated in the Brazilian trial within 90 days from stroke onset; National Institute of Health stroke scale (NIHSS) score was ≥8 at inclusion in the Spanish trial and 4 to 20 in the Brazilian trial; and age inclusion criteria had the upper limit in 80 years in the Spanish trial and 75 years in the Brazilian trial.

In both trials patients with lacunar or hemorrhagic stroke were excluded. Other exclusion criteria were pregnancy, history of neoplasia, life threatening illness, hematological diseases, significant previous disability (prestroke modified Rankin Scale [mRS] score ≥3), and severe comorbidity (severe hepatic or renal dysfunction) that would preclude follow-up.

Treatment and outcome assessment were done according to the individual study protocols that were approved by the relevant institutional review boards. Written informed consent was obtained from each patient or their representatives. The trials were registered with clinicaltrials.gov (trial identification numbers NCT00761982 [7] and NCT00473057 [8, 9]).

### 2.2. Cell Therapy Procedures

Transplantation procedure was done in the BM-MNC-treated group as previously described [7, 8]. In brief, 50–80 milliliters of bone marrow was obtained by puncture in the posterior iliac crest. The aspirate was centrifuged on a Ficoll density gradient to isolate the mononuclear cells, which were injected in the M1 segment of the infarct-related MCA in approximately 10 minutes. In five patients in the Brazilian trial, BM-MNCs were administered intravenously into the antecubital vein. In every patient, the injection was performed approximately at the rate of 1 mL/min. No bone marrow aspiration or sham injection was performed in the control group.

### 2.3. Outcomes Evaluation

Clinical and functional evaluation (m-Rankin Scale and NIHSS) were performed after transplantation and 1, 3, and 6 months after the stroke.

In the pooled analysis, the primary outcome measure was the score on the mRS at 6 months dichotomized between good outcome (mRS 0 to 2) and unfavourable outcome (mRS 3 to 6).

Modified Rankin Scale score measures functional outcome after stroke [21]. Scores range from 0 to 6: 0 indicating no symptoms at all; 1 indicating no significant disability despite symptoms, being able to carry out all usual duties and activities; 2 indicating slight disability, being unable to carry out all previous activities but able to look after own affairs without assistance; 3 indicating moderate disability, requiring some help, but being able to walk without assistance; 4 indicating moderately severe disability, being unable to walk without assistance and unable to attend to own bodily needs without assistance; 5 indicating severe disability, being bedridden, incontinent, and requiring constant nursing care and attention; and 6 indicating death.

### 2.4. Statistical Analysis

The Kolmogorov-Smirnov test was applied to verify if the variables followed a normal distribution. When the variables were not normally distributed, comparisons between groups were made using the Mann-Whitney* U* tests to detect differences in the distribution of samples and Spearman's Rho coefficient to assess the relationship between two quantitative variables. Categorical data are expressed as percentages and analyzed using the Chi-square test (*χ* ^2^) or Fisher's exact test where appropriate. Spearman's rank correlation coefficient was used to assess the association between outcomes (mRS and NIHSS) and the number of BM-MNCs injected. The number of BM-MNCs injected was dichotomized at the best cut-off point for a better accuracy in predicting good outcome at six months using receiver operating characteristics (ROC) curves. All statistical analyses were performed using the SPSS software package version 18.0 for Windows (SPSS Inc., Chicago, IL, USA). Differences were considered to be statistically significant when two-tailed *p* values were less than 0.05.

## 3. Results

Of the 22 patients treated with BM-MNCs, 68.2% were men and mean age was 60.4 years (±14). Main risk factors were hypertension (68.2%), diabetes (40.9%), and dyslipidemia (40.9%). All have a moderate-to-severe MCA stroke at inclusion with a median NIHSS score of 13.0 (IQR 9.75–16.9). Cardioembolic stroke was the most frequent mechanism of stroke (40.9%) and intravenous thrombolysis was performed in the acute phase of stroke in 31.8% of patients (Table 1).

Both cohorts were very similar in baseline characteristics except for age (67.4 years of age in those patients included in the Spanish trial versus 54.6 in the Brazilian trial, *p* = 0.03) and days from stroke to treatment (BM-MNCs injection at 6.3 days from stroke onset in the Spanish trial versus 58 days in the Brazilian trial, *p* < 0.001).

A median of 153 × 10^6^ (±121 × 10^6^) BM-MNCs was injected in the 22 cases. Intra-arterial route was used in 17 patients (77.3% of cases).

Regarding safety, during follow-up there was no deaths, tumor formation, or stroke recurrence. Seven patients (31.8%) had a partial seizure during follow-up and 4 patients (18.2%) had a systemic infection (Table 2).

**Figure 1:**
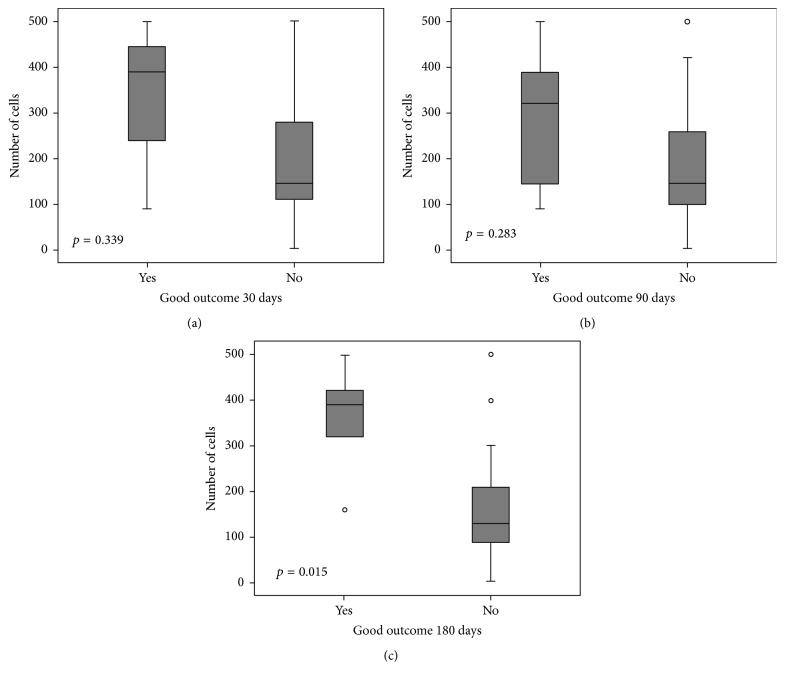
Box plot of the relation between number of injected cells and stroke outcomes during follow-up. Patients with good outcomes had no or mild disability (mRS of 0–2). Values are expressed in millions of cells. Small circles mean outliers.

When evaluating dose of BM-MNCs administered, the higher number of injected cells was associated with better outcomes at 180 days (390 × 10^6^ [320–422] BM-MNCs injected in those patients with mRS of 0–2 versus 130 × 10^6^ [89–210] in those patients with mRS 3–6 at 6 months, *p* = 0.015) (Figure 1).

There were no significant correlations between number of cells and NIHSS or mRS at any time point when analyzing all the BM-MNC-treated patients included in the study (*n* = 22), but there was a trend towards less disability when a higher number of cells were injected (mRS at 90 days (*r* = −0.372, *p* = 0.088) and mRS at 180 days (*r* = −0.389, *p* = 0.074)). However, when analyzing only those patients treated intra-arterially with BM-MNCs (*n* = 17), a strong negative correlation between dose of cells and mRS score at 6 months was found (*r* = −0.63, *p* = 0.006). Also, there was a significant relationship between dose of intra-arterial BM-MNCs and disability in follow-up (390 × 10^6^ [240–461] BM-MNCs injected in those patients with mRS of 0–2 at 6 months versus 121 × 10^6^ [79–208] in those patients with mRS 3–6, *p* = 0.009). The low number of patients treated intravenously (*n* = 5) did not allow a separate analysis of the relationship between number of cells injected and outcomes in this subgroup of patients.

**Figure 2:**
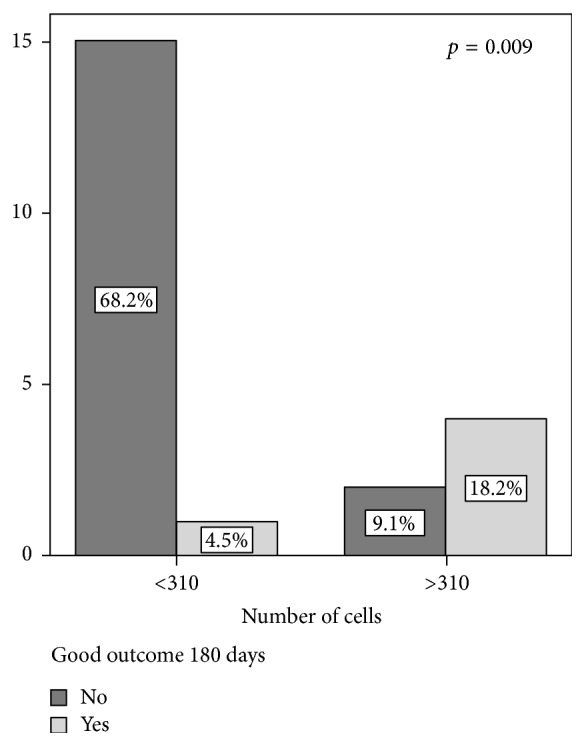
Analysis of a dose cut-off (310 × 10^6^ BM-MNCs injected) and outcomes after 180 days. Patients with good outcomes had no or mild disability (mRS of 0–2). Values are expressed in millions of cells.

When analyzing receiver operating characteristics (ROC) curves, a cut point of 310 × 10^6^ cells injected predicted a good outcome, with no or mild disability at 180 days after stroke (mRS of 0–2) with a sensitivity of 80% and specificity of 88.2% (Figure 2).

## 4. Discussion

This pooled analysis of two different clinical trials with autologous BM-MNCs injection in ischemic stroke patients gives some light about optimal doses of cells to be tested in clinical trials, especially when intra-arterial route is used. To the best of our knowledge, we describe for the first time a significant relationship between higher dose of BM-MNCs and better outcomes in stroke patients and a possible efficacy dose cut-off for cell therapy trials.

This relationship is plausible as in preclinical studies there is strong evidence of the importance of cell dose in neurological outcomes after brain ischemia. In a recent meta-analysis of preclinical studies with mesenchymal stem cells (MSCs) for cerebral ischemia, authors found a negative correlation between dose of cells and neurological deficit during follow-up (*r* = −0.63, *p* < 0.001) [13], similar to our findings.

Since preclinical data indicate that dose is an important factor in optimizing cell therapy, low doses might not improve functional outcomes and larger number of cells might be unnecessary [22]. Our data indicates that the optimal threshold of transplanted cells is probably around 310 × 10^6^ BM-MNCs in order to obtain good functional outcome with high probability amongst treated stroke patients.

However, available data about dose of cells in humans is conflicting. In line with our results, Taguchi et al. evaluated in a clinical trial two different doses of BM-MNCs administered intravenously in stroke patients after 7–10 days of stroke onset (250 × 10^6^ and 340 × 10^6^ cells in the lower and higher dose groups, resp.) and although it was a phase I/IIa clinical trial not designed to test efficacy, authors described a trend towards improved neurological outcomes in those patients receiving the higher dose of bone marrow cells [20].

On the other side, Prasad et al. published a phase II trial including 120 stroke patients with fifty-eight of them being treated with intravenous injection of BM-MNCs, showing no relationship between cell dose and outcomes [10].

Also, in a meta-analysis of cell-based therapies for treating stroke patients [23], authors found that stem cell therapy was more effective with higher dose of cells and also when intra-arterial route was used. In Table 3, published clinical trials with BM-MNCs in stroke patients are listed. Unfortunately, we were not able to obtain the information of other published BM-MNCs clinical trials and include them in the pooled analysis. Although the relevance of cell dose in clinical trials with stroke patients is not clear, regarding safety, there is no data in the literature about potential negative outcomes when higher BM-MNCs doses are injected. However, future trials testing different doses of cells should evaluate carefully not only efficacy but also the safety of higher doses of cells.

Regarding the route, most of the patients included in this pooling data study were treated using intra-arterial route. We found a trend towards less disability when higher number of cells was injected when all the patients included were analyzed. However, we found a strong negative correlation between cell dose and disability when intravenous patients were excluded from analysis, pointing to the hypothesis that the combination of higher number of cells and intra-arterial route could be a key factor to improve neurological outcomes in stroke patients.

Based on animal models of stroke it is not clear which route of delivery is preferable [13]. Intravenous cell delivery is a less invasive route and is increasingly used in clinical trials. However, preclinical studies have indicated that the intravenous injection leads to significant cell trapping in organs such as the lungs, liver, and spleen, with a small number of cells reaching the ischemic brain [12, 24]. Previous observations showed that delivery routes dramatically affect the migration and distribution of grafted cells and that administration of bone marrow-derived cells using more invasive methods such as intra-arterial route may provide significantly greater benefit in stroke [13, 24]. Despite the concern for the possibility of cerebral microembolism during intra-arterial injection leading to new focal ischemic lesions and worsening of neurological deficit, we did not detect new strokes or neurological deterioration after intra-arterial injection of BM-MNCs. Also, in preclinical studies it has been stated that vascular occlusion is related to the type of cells transplanted [25]. Although intra-arterial delivery of MSCs increased the risk of new ischemic lesions and mortality in rats [26], the intra-arterial injection of BM-MNCs was safe and did not reduce cerebral perfusion [5, 25, 27]. This may be related to the smaller size of BM-MNCs compared to MSCs [25].

Although the mechanisms underlying cell therapy recovery are unclear, a potential explanation of the relevance of dose could be the secretion of cytokines and growth factors by BM-MNCs [25]. These cytokines are involved in angiogenesis and neurogenesis but also can reduce proinflammatory response after stroke [5, 6]. In this line, our group described previously a significant negative correlation between number of cells injected and several cytokines such as MMP-2, playing a possible anti-inflammatory role and a positive correlation with GM-CSF and PDGF-BB, factors related to brain plasticity [28]. Therefore, we could hypothesize that higher number of cells transplanted could have greater neurorestorative effects in stroke patients through secretion of larger amount of some beneficial cytokines.

The main limitation of this study is that, due to the small sample size, logistic regression analysis could not be performed to evaluate the independent predictors of good outcome. However, our results are similar to the strong evidence previously described in stroke animal models and the meta-analysis of published clinical trials [23]. To confirm the relationship between cell dose and outcomes, a dose-finding multicenter clinical trial is ongoing in ischemic stroke patients treated with different doses of intra-arterial BM-MNCs [29].

## 5. Conclusions

In conclusion, higher dose of autologous BM-MNC seems to be related to less disability in ischemic stroke patients, similar to preclinical studies, especially when more than 310 × 10^6^ cells are injected. This relationship seems to be stronger when injection is performed intra-arterially. Further interventional studies are warranted to confirm these data.

## Figures and Tables

**Table 1:** Baseline characteristics of patient treated in both clinical trials.

	All (*N* = 22)	Spanish trial (*N* = 10)	Brazilian trial (*N* = 12)	*p*
Age	60.4 ± 14	67.4 ± 13	54.6 ± 13	0.03^*∗*^
Gender (male)	15 (68.2%)	5 (50%)	10 (83.3%)	0.17
Hypertension	15 (68.2%)	6 (60%)	9 (75%)	0.62
Diabetes	9 (40.9%)	3 (30%)	6 (50%)	0.41
Dyslipidemia	9 (40.9%)	4 (40%)	4 (33.3%)	0.99
Tobacco	5 (22.7%)	1 (10%)	4 (33.3%)	0.32
Cardiopathy	2 (9.1%)	0 (0%)	2 (16.7%)	0.48
AF	4 (18.2%)	2 (20%)	2 (16.7%)	0.99
TOAST				
LAA	3 (13.6%)	0 (0%)	3 (25%)	0.39
CE	9 (40.9%)	4 (40%)	5 (41.7%)	
LAC	1 (4.5%)	1 (10%)	0 (0%)	
UND	7 (31.8%)	4 (40%)	3 (25%)	
OTH	2 (9.1%)	1 (10%)	1 (8.3%)	
Side				
Right	9 (40.9%)	4 (40%)	5 (41.7%)	0.99
Left	13 (59.1%)	6 (60%)	7 (58.3%)	
VB	—	—	—	
IV thrombolysis	7 (31.8%)	4 (40%)	3 (25%)	0.65
IA therapy	1 (4.5%)	0 (0%)	1 (8.3%)	0.99
NIHSS (baseline)	13.0 [9.7–16.0]	15.5 [10.7–18.0]	11.5 [9.0–14.5]	0.07
Infarct volume^#^	84.4 ± 65.5	62.0 ± 60.5	99.4 ± 66.9	0.22
Injection days	34.5 ± 32.4	6.3 ± 1.3	58 ± 26	<0.001^*∗*^
Number of cells	153.5 (100–320)	138.5 (76–210)	223.5 (128–395)	0.20

Values are expressed as means ± SD. ^*∗*^ *p* < 0.05; ^#^ *n* = 20. BM-MNC indicates bone marrow mononuclear cell.

**Table 2:** Follow-up of patients treated with BM-MNCs.

	All (*N* = 22)	Spanish trial (*N* = 10)	Brazilian trial (*N* = 12)	*p*
Death	—	—	—	—
Stroke	—	—	—	—
MI	—	—	—	—
Infection	4 (18.2%)	4 (40%)	0 (0%)	0.03^*∗*^
SICH	—	—	—	—
Allergic reaction	—	—	—	—
Tumors	—	—	—	—
Seizure	7 (31.8%)	2 (20%)	5 (41.7%)	0.38
NIHSS 30	9.0 [6.0–12.0]	9.0 [7.0–13.0]	10.0 [5.2–12.0]	0.80
NIHSS 90	6.5 [4.7–11.2]	6.5 [5.7–11.7]	7.5 [4.0–11.7]	0.77
NIHSS 180	6.0 [4.0–10.2]	6.0 [3.7–11.2]	8.0 [4.2–10.7]	0.67
mRS ≤ 2 30	3 (13.6%)	1 (10%)	2 (16.7%)	0.99
mRS ≤ 2 90	5 (22.7%)	1 (10%)	4 (33.3%)	0.32
mRS ≤ 2 180	5 (22.7%)	2 (20%)	3 (25%)	0.99

^*∗*^ *p* < 0.05.

**Table 3:** Published clinical trials with BM-MNC therapy in stroke patients.

Author	Cell population	Number of patients treated (controls)	Design	Route	Cell dose	Time window	Follow-up (months)	Adverse events
Suárez-Monteagudo et al. [14]	Autologous	5 (0)	Open-label phase I	Intraparenchymal	1.4–5.5 × 10^7^	1–10 years	12	Headache, drowsiness, nausea, fever
Savitz et al. [11]	Autologous	10 (0)	Open-label phase I	Intravenous	7–10 × 10^6^/kg	24–72 hours	6	None study-related reported
Friedrich et al. [15]	Autologous	20 (0)	Open-label phase I	Intra-arterial	2.2 × 10^7^	3–7 days	6	None study-related reported
Moniche et al. [7]	Autologous	10 (10)	Observer-blinded phase I/II	Intra-arterial	16 × 10^7^	5–9 days	6	Seizures (2 of 10)
Prasad et al. [16]	Autologous	11 (0)	Open-label phase I	Intravenous	0.2–18 × 10^7^	7–30 days	6	One reinfarction
Li et al. [17]	Autologous	60 (40)	Observer-blinded phase I/II (hemorrhagic stroke)	Intraparenchymal	0.2–2 × 10^7^	5–7 days	6	Fever, one unspecified pulmonary tumor
Barbosa da Fonseca et al. [18], Battistella et al. [8], Rosado-de-Castro et al. [9]	Autologous	12 (0)	Open-label phase I	Intravenous (*n* = 5), intra-arterial (*n* = 7)	10–50 × 10^7^	19–89 days	6	Seizures (7 of 12)
Prasad et al. [10]	Autologous	85 (35)	Blinded randomized phase II	Intravenous	28 × 10^7^	18.5 days	12	None reported
Sharma et al. [19]	Autologous	24 (0)	Open-label phase I/II (ischemic and hemorrhagic stroke)	Intrathecal	1 × 10^6^/kg	40 months	30	None reported
Taguchi et al. [20]	Autologous	12 (0)	Open-label phase I/II	Intravenous	29 × 10^7^	7–10 days	6	One recurrent stroke
