# Corrigendum to “Increasing Dose of Autologous Bone Marrow Mononuclear Cells Transplantation Is Related to Stroke Outcome: Results from a Pooled Analysis of Two Clinical Trials”

**DOI:** 10.1155/2017/7946930

**Published:** 2017-08-13

**Authors:** Francisco Moniche, Paulo Henrique Rosado-de-Castro, Irene Escudero, Elena Zapata, Francisco Javier de la Torre Laviana, Rosalia Mendez-Otero, Magdalena Carmona, Pilar Piñero, Alejandro Bustamante, Lucía Lebrato, Juan Antonio Cabezas, Alejandro Gonzalez, Gabriel R. de Freitas, Joan Montaner

**Affiliations:** ^1^Department of Neurology, Hospital Universitario Virgen del Rocío, 41013 Seville, Spain; ^2^Instituto de Biomedicina de Sevilla-IBiS, Hospital Universitario Virgen del Rocío, 41013 Seville, Spain; ^3^Instituto de Ciências Biomédicas, Federal University of Rio de Janeiro, 21044-020 Rio de Janeiro, RJ, Brazil; ^4^Instituto de Biofísica Carlos Chagas Filho, Federal University of Rio de Janeiro, 21044-020 Rio de Janeiro, RJ, Brazil; ^5^Department of Hematology, Hospital Universitario Virgen del Rocío, 41013 Seville, Spain; ^6^Department of Radiology, Hospital Universitario Virgen del Rocío, 41013 Seville, Spain; ^7^Neurovascular Research Laboratory, Institut de Recerca Vall d'Hebron, Hospital Vall d'Hebron, 08035 Barcelona, Spain; ^8^D'Or Institute for Research and Education, 22281-100 Rio de Janeiro, RJ, Brazil

In the article titled “Increasing Dose of Autologous Bone Marrow Mononuclear Cells Transplantation Is Related to Stroke Outcome: Results from a Pooled Analysis of Two Clinical Trials” [1], the name of the thirteenth author was given incorrectly as Grabriel R. de Freitas. The author's name should have been written as Gabriel R. de Freitas. The revised authors' list is shown above.
